# Lakes in Hot Water: The Impacts of a Changing Climate on Aquatic Ecosystems

**DOI:** 10.1093/biosci/biac052

**Published:** 2022-07-18

**Authors:** R Iestyn Woolway, Sapna Sharma, John P Smol

**Affiliations:** Department of Meteorology, University of Reading, Reading, England and with the School of Ocean Sciences at Bangor University in Anglesey, Wales; Department of Biology, York University, Toronto, Ontario, Canada; Department of Biology, Queen’s University, Kingston, Ontario, Canada

**Keywords:** limnology, ecology, climate change, environmental science, water resources

## Abstract

Our planet is being subjected to unprecedented climate change, with far-reaching social and ecological repercussions. Below the waterline, aquatic ecosystems are being affected by multiple climate-related and anthropogenic stressors, the combined effects of which are poorly understood and rarely appreciated at the global stage. A striking consequence of climate change on aquatic ecosystems is that many are experiencing shorter periods of ice cover, as well as earlier and longer summer stratified seasons, which often result in a cascade of ecological and environmental consequences, such as warmer summer water temperatures, alterations in lake mixing and water levels, declines in dissolved oxygen, increased likelihood of cyanobacterial algal blooms, and the loss of habitat for native cold-water fisheries. The repercussions of a changing climate include impacts on freshwater supplies, water quality, biodiversity, and the ecosystem benefits that they provide to society.

There are more than 100 million lakes in the world  (Verpoorter et al. 2014), which hold approximately 87% of the planet's liquid surface freshwater (Gleick 1993) and provide multiple ecosystem services to people (Rinke et al. 2019). Lakes are critical resources for water supplies and food security (irrigation and fisheries), as well as highly valued destinations for recreation and tourism. Lakes are also recognized as key sentinels of climate change, which is reflected by a wide range of physical, chemical, and biological responses to climate variations and climate-induced changes within the lake catchment (Adrian et al. 2009, Williamson et al. 2009). Lake variables are therefore considered to be essential climate variables (Woolway et al. 2020)—that is, variables that critically contribute to the characterization of Earth's climate.

Lakes are changing rapidly in response to climatic shifts and have responded to both natural and anthropogenic stressors in recent decades. Climate is the big threat multiplier in lakes (Smol 2010), often magnifying environmental stressors such as eutrophication, cross-boundary contamination, and the spread of invasive species (Moss et al. 2011, Birk et al. 2020). This is particularly apparent in more than half the world's lakes that experience seasonal ice cover (Hampton et al. 2017). One of the most realized consequences of climate warming in lakes is an earlier and more prolonged loss of winter ice, which has been noted in recent decades (Imrit and Sharma 2021, Sharma et al. 2021a) and often results in a cascade of ecological and environmental consequences, from disrupted trophic links to altered food webs (Hébert et al. 2021). Some of the largest ecological changes are occurring in northern high latitudes, where about one-quarter of our planet's lakes are situated (Lehner and Döll 2004). These aquatic ecosystems are considered to be especially sensitive bellwethers of environmental change (Smol 2016). However, climate change is also affecting other types of lakes in diverse ways. For example, in historically ice-free, cool lakes, such as some well-studied sites in the Ecuadorian Andes, historically polymictic systems are now thermally stratified for extended periods of time (Michelutti et al. 2015, Michelutti et al. 2016, Labaj et al. 2018). In ice-free, warm tropical lakes, thermal stratification has become more intense (Cohen et al. 2016). Although some of these consequences have been discussed previously, often in isolation, in the present article, we provide a synthesis that addresses the cumulative impacts and complex repercussions of climate change on lakes worldwide.

## A changing climate

Climate change is a threat multiplier that has profound implications for people around the world (Smol 2010). Surface air temperature is one of the most recognized indicators of climate change, with global observations suggesting that temperatures have risen by more than 1 degrees Celsius (°C) since the late twentieth century (GISTEMP Team 2021). Climate, however, is much more than temperature, and studies have recorded changes in a number of other essential climate variables, including a decadal-scale global increase in specific humidity—the amount of water vapor in the atmosphere—since the 1970s (0.07 grams  per kilogram per decade) and, over the same time interval, a decline in relative humidity (–0.09 % per decade; Willett et al. 2020). Also widely observed were multidecadal variations in solar radiation, often referred to as patterns of dimming and brightening, associated with air pollution patterns governed by economic development and air pollution regulations (Wild et al. 2005, Wild 2012). Decadal-scale variations in near-surface wind speed (Roderick et al. 2007, Vautard et al. 2010, McVicar et al. 2012, Zeng et al. 2019), including periods of stilling (–0.08 meters per second per decade from 1978 to 2010) followed by recovery (0.24 meters per second per decade from 2010 to 2017), were also observed (Zeng et al. 2019), likely influenced by decadal ocean–atmosphere oscillations and potentially related to higher surface roughness caused by vegetation growth or urbanization. Furthermore, an increase in the frequency and intensity of storms has been suggested (Rahmstorf and Coumou 2011, Seneviratne et al. 2012), including but not limited to an increase in both high wind and precipitation events in some regions, with an increase in drought conditions in others.

## How are lakes responding to climate change?

**Figure 1.**
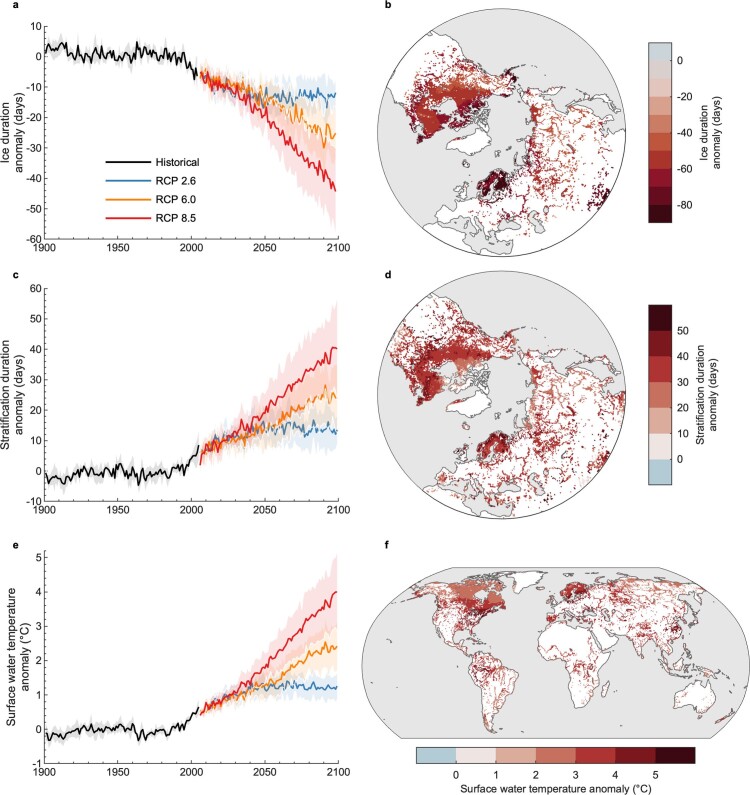
Historic and future projections of lake ice cover, thermal stratification, and water temperature. Temporal and spatial variations in (a, b) the duration of winter ice cover, (c, d) the duration of summer stratification, and (e, f) annually averaged surface water temperature. The temporal changes are shown (a, c, e) from 1901 to 2099 under historic and future climate forcing. The future simulations include three climate change scenarios: representative concentration pathway (RCP) 2.6 (low-emission scenario where emissions start declining at around 2020), 6.0 (medium-high-emission scenario where emissions peak at around 2080 and then decline), and 8.5 (high-emission scenario where emissions continue to rise throughout the twenty-first century). The thick lines show the average across numerous lake-climate model projections, and the shaded regions represent the standard deviation across the model ensemble. Panels (b), (d), and (f) show the spatial patterns by the end of the twenty-first century (averaged over all years from 2070 to 2099) under RCP 8.5. Anomalies are quoted relative to the 1970–1999 average (e.g., a positive value in panel (c) represents warmer conditions than estimated during the period 1970–1999). Source: The projections of lake ice cover and water temperature are from Grant and colleagues (2021), and the lake stratification projections are from Woolway and colleagues (2021b).

In response to climate change, lakes in the Northern Hemisphere are experiencing shorter winters, with accelerated losses of ice cover in the past few decades. Ice duration has declined by 31 days, on average, over the past 165 years (Woolway et al. 2020), with rates of ice loss six times faster in the past 25 years (Sharma et al. 2021a), and approximately 15,000 lakes in the Northern Hemisphere that historically froze every winter are now experiencing ice-free years (Sharma et al. 2019). These trends in ice loss are forecasted to continue with climate warming (Grant et al. 2021, Li et al. 2021). Many lakes may lose an additional 10–40 days of ice cover (figure 1) and approximately 5700 lakes may permanently lose ice cover this century (Sharma et al. 2021b). Because lakes inversely stratify under ice (Bruesewitz et al. 2015, Yang et al. 2020, Woolway et al. 2021c), a decrease in the duration of ice cover also leads to a shortening of the inversely stratified period (Woolway et al. 2021c). This is also evident in some ice-free lakes where surface water temperatures cool below approximately 4°C, the temperature of maximum density of freshwater (Woolway et al. 2019). In Northern Hemisphere lakes that inversely stratify, the duration of winter stratification is projected to shorten by an average of 18.5–53.9 days by the end of the twenty-first century under representative concentration pathways 2.6–8.5 (Woolway et al. 2021c). Moreover, as the ice-covered period shortens, the open-water season lengthens, and many lakes are projected to experience an earlier onset and longer periods of stable direct thermal stratification (Woolway et al. 2021b). By the end of the century, summer stratification is projected to begin up to 22.0 days earlier and end up to 11.3 days later in lakes throughout the Northern Hemisphere (Woolway et al. 2021b) and to be prolonged by between 10 and 35 days, depending on the future climate change scenario (figure 1). Earlier onset of thermal stratification has many limnological implications, including warmer summer water temperatures (Austin and Colman 2007, Woolway and Merchant 2017, Zhang et al. 2021) and cooler bottom temperatures in some (Bartosiewicz et al. 2019) but not all (Pilla et al. 2020) lakes. Lakes worldwide warmed at a rate of 0.34°C per decade between 1985 and 2009, with ice-covered lakes warming twice as fast as the global average (O'Reilly et al. 2015). Global average water temperatures may warm by an additional 1–4°C by the end of the century (Woolway and Maberly 2020, Grant et al. 2021) and may experience more extreme heatwaves (Woolway et al. 2021a), which will make it difficult for many freshwater taxa to find suitable thermal habitats (Kraemer et al. 2021).

Changes in the thickness and duration of the ice and snow cover on a lake will profoundly influence multiple key limnological properties. At high latitudes, such as in the High Arctic, where there are 24 hours of daylight in summer, these changes will be magnified. For example, with recent warming, some deep polar lakes, which, until recently, maintained a summer ice cover, are now ice free during the warm season, leading to numerous physical, chemical, and biological consequences (Lehnherr et al. 2018, Zhang et al. 2021). Two variables that have changed with declining ice and snow cover and that are apparent because of phenological shifts in ice-free stratified lakes (Woolway et al. 2021c) are the quantity and quality of light available for photosynthesis. Whereas most summertime photosynthesis in otherwise deeply frozen lakes was previously mainly restricted to open water in the littoral zone, with warming and complete seasonal ice loss, the entire lake surface receives sunlight, with consequent changes in lake biology (Smol and Douglas 2007a).

With continued warming, lakes that were historically mixed may also begin to display thermal stratification, crossing another key limnological threshold that affects mixing regimes and the lake biota (Rühland et al. 2015). Indeed, alterations in lake mixing regimes are projected this century, with many lakes typically—although not always—shifting to the right along the amictic–polymictic–dimictic–monomictic–oligomictic–meromictic continuum (Shatwell et al. 2019, Woolway and Merchant 2019). Climate change is expected to shift polymictic lakes to a monomictic regime and dimictic lakes to a monomictic or even to an oligomictic regime (Ficker et al. 2017, Vinnå et al. 2021). In some amictic and cold monomictic lakes, one might also expect an increase in the number of mixed days, because of declining lake ice cover. Of course there are some exceptions, with, for example, lakes shifting to the left along the mixing regime continuum (e.g., monomictic to polymictic) in regions in which wind speed is increasing (Woolway and Merchant 2019) or those influenced by increased glacier inflow (Peter and Sommaruga 2017) within a warming world.

## How does climate change affect water quantity?

**Figure 2.**
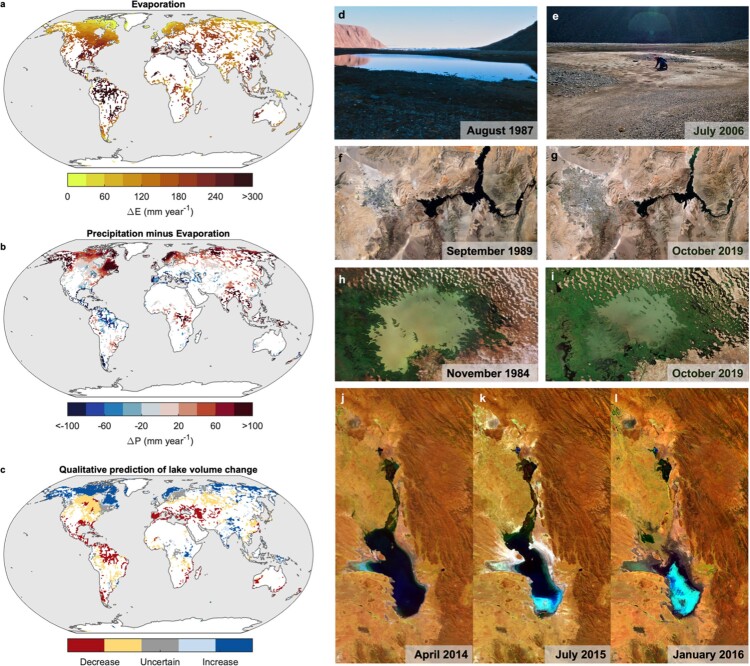
Projected and observed change in water quantity. Shown are simulated future changes (2071–2100 relative to 1971–2000) in (a) lake evaporation, (b) land P–E (precipitation minus evaporation), and (c) lake volume (based on the P–E balance over land and lake surfaces), under representative concentration pathway (RCP) 8.5. Model projections are from Zhou and colleagues (2021). Shown in panels (d) and (e) are what were previously permanent High Arctic ponds (Cape Herschel, Canadian High Arctic), that are now evaporating because of climate change. Pictured here is one of these sites, which used to be more than 100 meters long in the 1980s (d) but, since 2005, is dry by mid-July (e). Source: Reproduced with permission from Smol and Douglas (2007b). (f, g) Satellite images of Lake Mead (United States) taken on 2 September 1989 (Landsat) and 18 October 2019 (Sentinel 2), respectively, showing the decline in lake level or extent, as well as the expansion of Las Vegas to the west. (h, i) Satellite images of Lake Chad (bordering Chad, Cameroon, Niger, and Nigeria), taken on 5 November 1984 (Landsat) and 31 October 2018 (Sentinel 2). (j-l) Satellite images acquired from ESA's Proba-V minisatellite of Lake Poopó (Bolivia) acquired on 27 April 2014, 20 July 2015, and 22 January 2016, respectively. Images: Panels (f)–(i) contain modified Copernicus Sentinel data, processed by the ESA, CC BY-SA 3.0 IGO. Panels (j)–(l) contain modified satellite images from ESA/Belspo, produced by VITO.

By removing the lid of ice, the evaporation rates in lakes can increase markedly, with implications for water availability. Notably, as the duration of winter ice cover decreases in the near future and the physical barrier between the lake surface and the atmosphere is removed, water loss via evaporation will likely increase (figure 2; Wang et al. 2018). By the end of this century, the global mean annual lake evaporation is expected to increase by 16%, and at a rate of approximately 4% per degree increase in global-mean surface air temperatures (Wang et al. 2018). The largest increases in annual evaporation are expected at low latitudes, where evaporation rates are already high (Wang et al. 2018), but also in lakes that will transition to becoming ice free, allowing the the potential for evaporation to occur year-round (Woolway et al. 2020). Moreover, lake evaporation is expected to increase rapidly in regions that will experience a drying hydroclimate, which will amplify the evaporation increase by enlarging the surface water pressure deficit and reducing cloud shortwave reflection (Zhou et al. 2021). In the absence of substantial water inflow changes, the amplified evaporative loss will likely reduce lake volumes (Zhou et al. 2021) and, in turn, the quantity of freshwater (figure 2).

An often more important stressor for water quantity in lakes is direct human action, with surface water use for agriculture being the primary culprit for water scarcity in many regions (Pekel et al. 2016). Notable examples of water stress due to direct human action include the long-term decline of water level in Great Salt Lake, in the United States (Wurtsbauch et al. 2017), or the widely documented desiccation of the Aral Sea, in Kazakhstan and Uzbekistan (Micklin 1992, Micklin 2016, Wang et al. 2020), once the fourth largest lake in the world, caused primarily by withdrawals of water for irrigation. When combined with higher rates of evaporation or with a decrease in precipitation, surface-water use can greatly contribute to reducing the quantity of water in lakes (Schulz et al. 2020). Water level declines can be particularly severe in some regions, such as in Lake Chad (whose shores are in Chad, Cameroon, Niger, and Nigeria), historically ranked as one of the largest lakes in Africa, where lake level or extent has declined considerably because of decreases in local precipitation and discharge from its catchment, as well as increased evaporation (figure 2), or in Lake Poopó (Bolivia), where the withdrawal of water from the Desaguadero River, combined with anomalously low precipitation and high evaporation, contributed to lake levels reaching historic lows in 2015 and 2016 (figure 2; Torres-Batlló et al. 2020).

The effect of changing ratios of evaporation to precipitation, which exert primary control on water-level fluctuations in many lakes, particularly those that are shallow (e.g., Lake Poopó), has been suggested to result in some previously permanent water bodies now becoming ephemeral and some previously ephemeral water bodies now being completely dry (Finger-Higgens et al. 2019). Such changes have been widely reported in shallow polar ponds (Smol and Douglas 2007b), as well as in small saline systems located in arid or semiarid regions (Riveros-Iregui et al. 2017). Notably, with little or no surface water outlet, shallow saline lakes can undergo disproportionately large changes in water level and surface extent in response to small changes in precipitation or evaporation (Micklin 1992). As water levels drop in these lakes, salinity will increase further (Hammer 1986), with implications for the community composition, biomass, and the diversity of phytoplankton, zooplankton, macrophytes, and fish (Jeppesen et al. 2015). Increases in salinity caused by greater evaporation have also been observed in other lake types situated across climatic gradients, from subtropical (Hammer 1986) to High Arctic (Smol and Douglas 2007b, Paul et al. 2010) systems. In populated regions, salt application for deicing roadways is recognized as a major cause of salinity increase in lakes (Likens and Buso 2009, Müller and Gächter 2011, Swinton et al. 2014, Dugan et al. 2017) and will likely increase further in many northern regions under conditions of a warming world. Although it seems paradoxical to suggest that deicing roadways will become more common as the world warms (one might expect the opposite), salt is most effective at deicing roadways when air temperatures are above approximately –10°C but less effective at lower temperatures. Therefore, as winter air temperatures above this threshold prevail for longer periods of time, more road salt will likely be used. An increase in salinity may also influence the physical environment of lakes—notably, by altering the vertical water density gradient and increasing the stability of stratification or influence seasonal mixing (Boehrer and Schultze 2008, Ladwig et al. 2021). By altering the timing of seasonal stratification and mixing and, therefore, the duration of summer stratification, salinity changes in lakes represent a positive feedback on water level, which should be carefully considered in future climate change impact studies.

An alternative potential influence of climate change on lake level is evident in glacial lakes—particularly, closed-basin lakes in endorheic regions, where an increase in fresh glacial meltwater in recent decades could lead to higher lake levels (Shugar et al. 2020, Stuart-Smith et al. 2021). Increases in water quantity are also observed in some High Arctic systems, where thawing permafrost is resulting in the formation of thermokarst lakes, which can also be enhanced by local increases in precipitation (Rodell et al. 2018). Initial lake expansion may, however, give way to subsequent lake drainage as the surrounding permafrost degrades further with climate warming (Smith et al. 2005, van Huissteden et al. 2011). In general, lake area in the Arctic has increased in regions with permanent permafrost and decreased in regions where permafrost is thinner and less continuous (Smith et al. 2005).

## How does climate change influence water quality?

**Figure 3.**
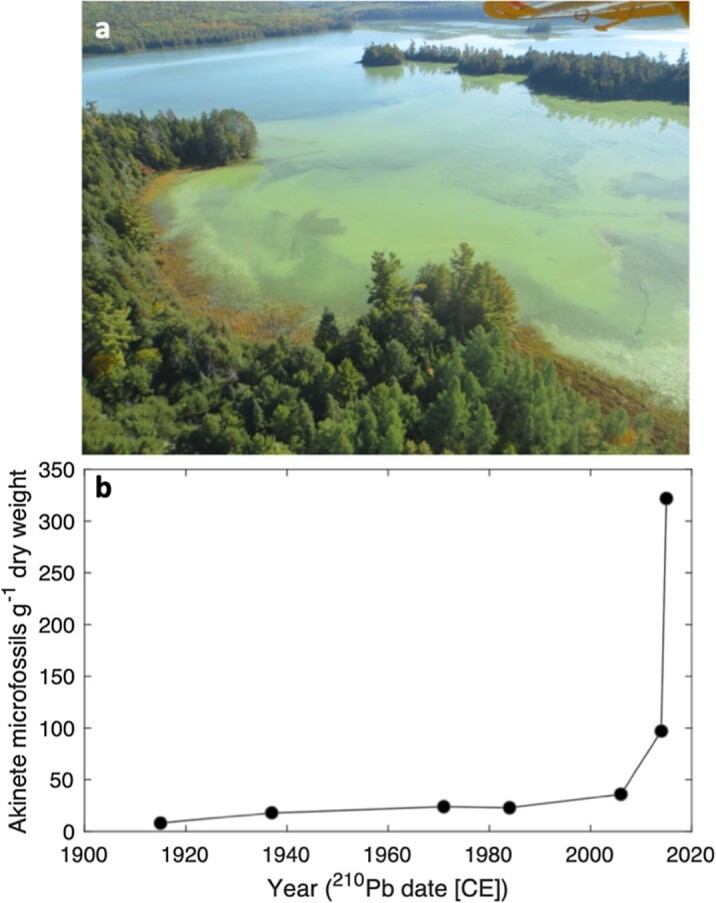
Cyanobacterial bloom in Dickson Lake (Ontario). (a) Aerial view of the Dolichospermum bloom on remote Dickson Lake (Algonquin Provincial Park, Ontario) in September 2014. Photograph: Courtesy of Alison Lake, Ontario Parks. (b) Concentrations of cyanobacterial akinetes (resting stages) in a high-resolution sediment core collected from Dickson Lake, scaled by age of sediment estimated from ^210^Pb dating, showing the unprecedented increases in cyanobacteria in the most recent sediments. Source: Redrawn from data presented in Favot and colleagues (2019).

Long-term changes in the climate, in addition to the increased frequency and magnitude of heatwaves and storms (i.e., that cause rapid nutrient delivery to lakes), as well as land-use changes, can combine to influence lake water quality. For instance, by delivering nutrients to surface waters, industry, agriculture, and urbanization has contributed to a well-documented increase in summer phytoplankton blooms (often used as a proxy for poor water quality) in many but not all (Oliver et al. 2017, Paltsev and Creed 2021, Topp et al. 2021, Wilkinson et al. 2021, Woolway et al. 2021d) lakes in recent decades (Ho et al. 2019, Hou et al. 2022). The most pronounced increases in lacustrine algal blooms have been suggested to occur in developing countries that remain reliant on agricultural fertilizer (Hou et al. 2022). When combined with warmer and longer summer stratification, conditions that favor bloom-forming algae in eutrophic lakes (i.e., those enriched with nutrients and minerals), water quality deterioration can be even more severe (Paerl and Huisman 2008). This may not be the case in nutrient-poor lakes, because stronger stratification can impede the entrainment of nutrients from deep water into the surface layer (Yankova et al. 2017, Schwefel et al. 2019, Lau et al. 2020). However, climate change, through direct and indirect temperature effects, can boost the development of harmful cyanobacterial (i.e., blue-green algal) blooms in both eutrophic and oligotrophic lakes (Reinl et al. 2021). These blooms rank among some of the main causes of poor water quality issues and can lead to mass mortalities of fish and birds, as well as providing a serious health threat for cattle, pets, and humans. For example, in Lake Taihu, in China, where algal blooms are widely reported, an earlier onset and a longer duration of *Microcystis* blooms have been attributed to earlier and warmer springs (Deng et al. 2014). In Ontario, Canada, not only have reports of algal blooms increased, but there are reports of such blooms as late as November, in response to climate warming, greater nutrient inputs, and increased public awareness (Winter et al. 2011). There have even been reports of cyanobacterial blooms in remote northern oligotrophic lakes (Pick 2016). For example, Dickson Lake, an oligotrophic, wilderness lake in Algonquin Provincial Park, Ontario, which is accessible only by hiking trails or float planes, began reporting large cyanobacterial blooms of the genus *Dolichospermum* in the autumn of 2014, which prompted park managers to ban overnight camping because of health concerns. Given the lack of direct long-term monitoring data, Favot and colleagues (2019) instigated a multidisciplinary paleolimnological study to determine whether these blooms were, in fact, unprecedented in recent history and whether any environmental triggers could be identified. Among the many paleoenvironmental proxies investigated, the concentrations of *cyanobacterial* akinetes (resting stages) in a ^210^Pb-dated high-resolution sediment core corroborated reports that the onset of blooms began in approximately 2014, and no comparable blooms had occurred in at least the last century (figure 3). Furthermore, the onset of blooms was consistent with warmer air temperatures, sharp declines in wind speed, and a lengthening of the ice-free season. With episodic storm events, the rapid delivery of nutrients into lakes can further drive early onsets of cyanobacterial blooms by increasing nutrient loading (Carpenter et al. 2018, Larsen et al. 2020). For example, the largest rainfall event ever recorded in the Grand River Watershed, Ontario, resulted in an increase of 7 to 27 times the bioavailable phosphorus, initiating an earlier bloom of toxic cyanobacteria, thereby elongating the season during which these toxic blooms may form (Larsen et al. 2020).

Blooms of cyanobacteria can become particularly thick in some lakes, resulting in a decrease in the amount of sunlight that reaches deeper waters. With less sunlight, photosynthesis in lakes is reduced, ultimately leading to a decrease in the concentration of dissolved oxygen available to support aquatic life. A decrease in dissolved oxygen can also occur via the deep-water decomposition of organic matter associated with blooms. When combined with an increase in water temperature, which leads to lower dissolved oxygen concentrations because of reduced gas solubility, a widespread pattern of lake deoxygenation emerges (Jane et al. 2021). In lakes that seasonally stratify, deep (hypolimnetic) waters will also likely experience anoxia (i.e., depleted of dissolved oxygen) as a consequence of stronger and more prolonged stratification, which, in many lakes, will exaggerate the severity of deoxygenation caused by eutrophication (Moss et al. 2011). The effects of eutrophication and climate change on deoxygenation will likely be worsened during the extreme warm years expected with climate change, as was observed during the extreme heatwave in the summer of 2003 in central Europe (Jankowski et al. 2006). In seasonally stratifying lakes, dissolved oxygen concentrations at depth are typically replenished during periods of water-column mixing. However, as lakes stratify for progressively longer periods—and, in extreme cases, transition to entirely different mixing regimes (e.g., from warm monomictic to oligomictic in deep tropical lakes)— replenishment of deep-water oxygen will occur at longer intervals or cease to occur altogether. This will have a positive feedback effect on water quality, as was described above, with deoxygenation at depth potentially leading to an increase in internal nutrient loading (when nutrients are introduced into the water column from the lake sediment) and, therefore, contributing to the formation of cyanobacterial blooms (Orihel et al. 2017, Favot et al. 2019, Molot et al. 2021, Smucker et al. 2021).

## Aquatic species in hot water

Warmer water temperatures, earlier onset and longer periods of thermal stratification, combined with lower dissolved oxygen concentrations can have important cumulative and potentially negative effects on aquatic organisms, such as fish. Climate warming is expected to benefit ectotherms by lengthening the summer growing season, particularly at high latitudes where it is historically short. Warmer summers are particularly beneficial to warm-water and nonnative generalist fishes (Sharma et al. 2007, Armstrong et al. 2021). For example, in the Arctic, lake trout productivity is projected to increase under future warming (Campana et al. 2020), and so are the growing conditions for juvenile salmon in southwestern Alaska (Schindler et al. 2005). Moreover, in Alaskan streams, warming temperatures have been suggested to increase overall community richness and abundance (Murdoch et al. 2020).

Climate change can also have numerous negative consequences for aquatic species; for example, declines in reproductive success following short and warm winters may counter the positive effects induced by warmer conditions (Farmer et al. 2015). Furthermore, an earlier start to summer can cause phenological mismatches in critical activities, such as spawning and foraging, often with widespread ramifications across the food web (Winder and Schindler 2004, Thackeray et al. 2010, Farmer et al. 2015, Dahlke et al. 2020, Hébert et al. 2021). Fish life stages most sensitive to temperature changes in the earlier part of the open-water season include embryos and spawning adults. Therefore, climate warming presents a critical bottleneck in the life history of some fish taxa (Dahlke et al. 2020). For example, egg survival of Arctic charr (*Salvelinus alpinus*) is limited by warming winter water temperatures, which already exceed tolerance temperatures in some European lakes (Kelly et al. 2020). Warming water temperatures and low oxygen concentrations at depth can also reduce the ecological niche dimensions of many fishes. Lake thermal habitats have already changed in response to warmer water temperatures and are forecast to change faster than the ability of some fish species to migrate to cooler regions (Woolway and Maberly 2020). Fish in large, tropical lakes are at the highest risk of extinction (Comte and Olden 2017, Kraemer et al. 2021). Such lakes often contain high fish diversity, with many taxa endemic to the lakes. Resident taxa are experiencing the highest amount of thermal habitat alterations (Kraemer et al. 2021). In temperate lakes, cold-water fishes are most vulnerable to being squeezed out of their preferred oxythermal habitats as summers become longer, because such fish require cold temperatures and high oxygen concentrations to thrive (Magnuson et al. 1985, Hasler et al. 2009, Jacobson et al. 2010, Magee et al. 2019, Till et al. 2019). For example, because of reduced access to suitable oxythermal habitats during warmer summers, lake trout were unable to access large prey from the littoral zone and, instead, ate smaller prey from pelagic regions, therefore experiencing reduced growth and poorer body condition (Guzzo et al. 2017). Novel competitive interactions caused by the invasion of warmwater fishes, such as smallmouth bass (*Micropterus dolomieu*), further exacerbate the stress that cool and cold-water fish face as water temperatures warm (Vander Zanden et al. 2001, Van Zuiden and Sharma 2016). In the Arctic, Pacific salmon (*Oncorhynchus* spp.) are undergoing a range shift with climate warming as higher numbers are being caught in Arctic waters (Dunmall et al. 2018). Although this may be construed to be a positive consequence of climate change for local communities that now have a new food source, the question remains as to how these range expansions will affect native fish populations, such as the Arctic charr, a species already stressed by climate warming (Kelly et al. 2020).

In contrast to many temperate and polar aquatic ecosystems with declining ice cover, where primary production is suggested to have increased because of climate change and a lengthening of the growing season (O'Beirne et al. 2017, Griffiths et al. 2017), primary production may be diminishing in many tropical lakes as nutrients are increasingly trapped at depth because of a strengthening of thermal stratification (Saulnier-Talbot et al. 2014, Cohen et al. 2016). For example, in deep (approximately 1470 meters) tropical Lake Tanganyika, paleolimnological evidence suggests that recent climate warming was unprecedented since at least 500 CE (Tierney et al. 2010) with important limnological repercussions (Verburg et al. 2003), including reduced ecosystem productivity (O'Reilly et al. 2003). Similarly, in high-altitude lakes of the Ecuadorian Andes, a switch from polymixis to thermally stratified conditions with post-1970s warming (Michelutti et al. 2016, Labaj et al. 2018) caused marked shifts in lake ecosystem function, including declining productivity, demonstrated using paleolimnological approaches (Michelutti et al. 2015). Climate-driven declines in lake primary production can cascade through the food web, ultimately affecting fish and other resources on which people depend. For example, Cohen and colleagues (2016) found that fish stocks in Lake Tanganyika, which began to decline well before the advent of commercial fishing, were linked to more intense warming over approximately the last 150 years.

## Ecosystem services

The implications of climate change for the ecosystem services and other benefits that lakes provide are numerous. Benefits include provisioning, regulating, supporting, and cultural services, each of which will be influenced by climate change. Cultural ecosystem services, which include aesthetics and recreation, will clearly be affected by climate change. For example, a decline in winter ice cover will influence recreation activities such as skiing, snowmobiling, and ice fishing, among others (Brammer et al. 2015, Knoll et al. 2019). The aesthetic appeal of lakes can be influenced by an increased prevalence of algae blooms that will, in turn, negatively affect tourism and lakeside property values (Clapper and Caudill 2014, Liu et al. 2017). Such blooms can lead to lake recreation closures (e.g., swimming, fishing), as occurred in the English Lake District (in the United Kingdom) in 2010. That year, the United Kingdom's biggest wild swimming event (the Great North Swim) was cancelled, with financial consequences for the local economy. Toxic algae blooms also negatively affect potable freshwater supplies, as occurred in Toledo, Ohio (in the United States), in 2014, when a cyanobacteria bloom in Lake Erie, caused water shortages and resulted in substantial costs for water managers. Also, in 2007, a massive toxic cyanobacterial bloom in Lake Taihu (in China) shut down the water supply for 2 million people for a week in Wuxi city (Duan et al. 2009).

A decline in the availability of safe drinking water caused by harmful algae blooms is considerably worse when combined with a reduction in water quantity. The latter can also have a profound impact on local economies by, for example, altering shipping channels, further impeding tourism and recreational activities, and increasing operational risks to industries that rely on lakes for heating and cooling purposes (Fink et al. 2014, Gaudard et al. 2019). Although the influences of climate change on the aforementioned ecosystem services are by no means easy to predict, they can at least be quantified, given the information available on past occurrences. However, some of the most difficult consequences to foresee, which may also have the most severe impact, are those associated with the influence of climate change, habitat degradation, water pollution, and invasive species on freshwater biodiversity, which is already estimated to have declined by 83% since 1970 (WWF 2018) because of habitat degradation, water pollution, and invasive species, all of which have unfolded because of human behaviors and land-use change. Climate change is now making its presence known as an additional driver or enhancer. The increase of warm-water and the decline of cold-water species and the subsequent introduction of invasive species can result in vast environmental and economic costs. For example, even before the turn of the millennium, it was estimated that invasive species caused more than US$120 billion worth of damages per year in the United States alone (Pimentel et al. 2000). More recently, it was estimated that costs associated with biological invasions worldwide between 1970 and 2017 amounted to at least US$1.288 trillion (Diagne et al. 2021).

## Future directions: Preparing for the perfect storm

**Figure 4.**
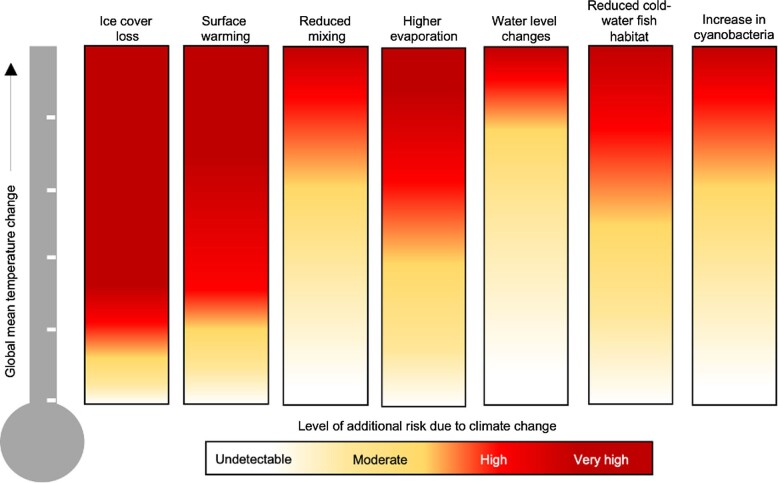
Expected climate change responses of key lake ecosystem processes. Shown are the level of additional risk to important physical, chemical, and ecological lake processes due to global warming, based on our expectations. White indicates an undetectable level of risk, meaning that lake ecosystems will be somewhat resilient to change under those specific levels of warming, and dark red illustrates a very high risk of ecosystem response. We stress that there will be exceptions to these expected changes, as is highlighted in our review.

Climate change has unquestionably altered lake ecosystems worldwide, and we expect the long-term patterns of change observed in recent decades to not only continue, but, in many cases, to worsen in the near future (figure 4). Interactions between climate and other aquatic ecosystem stressors will likely lead to unexpected, nonlinear ecological responses, resulting in more severe impacts on aquatic ecosystems than currently observed. Moreover, although the exceedance of temperature thresholds for many aquatic species, as well as regime shifts in the physical environment of lakes (e.g., mixing regime alterations and the total loss of winter ice cover), are already documented, these will likely become more prevalent in the future and, therefore, have widespread implications for lake ecosystems.

Nonlinear responses of lakes to climate change will also complicate the development of effective management strategies; the compounding influence of multiple stressors, in addition to currently unforeseen ecosystem responses, will result in unprecedented and unpredictable changes to aquatic ecosystems worldwide. Whereas individual lake ecosystem responses to climate change have typically been studied in isolation, such as warming of lake surface waters or depletion of water-column dissolved oxygen concentrations, when multiple stressors co-occur, the resulting perfect storm will have potentially dramatic consequences for lake ecosystems. In addition, many future ecosystem responses may not appear gradually but, rather, will occur quickly and often without warning, leaving little time for adaptation. One key area for future research is the development of early warning systems for such abrupt shifts. This should include development of more advanced, process-based aquatic ecosystem models that focus on the species level, and engagement of community scientists to identify early warning signals and use the wealth of data being accumulated from Earth Observation platforms and long-term monitoring networks, to identify critical transitions (Scheffer et al. 2009), but also the continued development of quantitative methods that can identify spatial hotspots for abrupt shifts and tipping points where time-series data are lacking. Ultimately, the myriad issues that affect lakes require both immediate and ongoing study to understand the complex responses of lake ecosystems to climate change.

Striking ecological changes in aquatic ecosystems have already been documented. Nevertheless, these impacts are probably only the tip of the iceberg. We should not expect gradual ecosystem responses to climate change, as is illustrated by global projections (i.e., figure 1), but, rather, tipping point responses that lead to new ecosystem states once ecological thresholds are crossed (Scheffer and Carpenter 2003). The ecological consequences of climate change will be abrupt, and the impacts of extreme climate events are already occurring and will continue to be felt locally, without warning, and without time to adapt. The effects of climate change often occur cumulatively and interact synergistically with multiple environmental stressors. Climate change will exacerbate problems with water quantity and quality, the latter including eutrophication, salinization, contamination, and the spread of invasive species, to name a few.

Water is a fundamental resource without which humans cannot survive. We must develop a better mechanistic understanding of the relation between climate change and lake function and recognize the early warning signals (e.g., winters without mixing in traditionally monomictic lakes or the presence of cyanobacterial blooms in oligotrophic lakes) of the consequences of climate change in freshwater ecosystems. Recent advances in technology, such as remote sensing and environmental DNA, combined with a move to work beyond traditional silos, provide an unprecedented opportunity to better understand lake responses to an uncertain future world. Notably, by integrating the strengths of remote sensing, *in situ* observations, traditional ecological knowledge, experiments, process-based modeling, and paleolimnology, we are uniquely positioned to uncover the impacts of climate change on lakes at broad spatial and temporal scales (Thackeray and Hampton 2020). If the global community wants to move toward equitable access to clean water by 2030, as was outlined in the United Nations’ Sustainable Development Goal 6.1, the inclusion of diverse voices from researchers worldwide, including the Global South, and the cross-pollination of ideas across disciplines, will be essential.

## Notes

R. Iestyn Woolway is affiliated with the Department of Meteorology at the University of Reading, in Reading, England and with the School of Ocean Sciences at Bangor University in Anglesey, Wales. Sapna Sharma is affiliated with the Department of Biology at York University, in Toronto, Ontario, Canada. John P. Smol is affiliated with the Paleoecological Environmental Assessment and Research Lab and with the Department of Biology at Queen’s University, in Kingston, Ontario, Canada.
